# Modification of the Mammalian Endomembrane System in Healthy and Diseased Cells

**DOI:** 10.3390/ijms21062133

**Published:** 2020-03-20

**Authors:** Jeremy C. Simpson

**Affiliations:** School of Biology and Environmental Science & Conway Institute of Biomolecular and Biomedical Science, University College Dublin, Dublin 4, Ireland; jeremy.simpson@ucd.ie

One remarkable characteristic of eukaryotic cells is the complexity of their membrane systems. Indeed, the evolution of distinct subcellular organelles, each with a defined set of biochemical functions, has been an essential feature in the specialisation of individual cells and cell types. Alongside the diversity of reactions that cellular compartments can carry out, another fascinating aspect is the variety of organelle morphologies that are seen, ranging from simple spherical profiles, through flattened sheets and tubules, to highly complex branched membrane networks. Not only does this high complexity of membrane architecture require the involvement of many proteins, lipids and carbohydrates for its maintenance, but it is becoming increasingly clear that in the diseased state, many endomembrane structures become modified. In this special issue, entitled ‘Molecular Regulation of the Endomembrane System’, a total of nine articles have been published that discuss aspects of the above phenomenon, providing insight into the key subcellular organelles found in eukaryotic cells (Table 1). 

Central to all of the articles is the role of local lipid composition, and particularly, that defined gradients of different lipid classes are found across subcellular organelles. The review by Casares and colleagues [6] summarises the main classes of lipids, including glycerolipids, phospholipids, sphingolipids and cholesterol, found in cells, and goes on to detail their relative abundance across subcellular structures. This article also provides insight into how these different lipids contribute to the regulation of organelle morphology through the different molecular shapes that they adopt. Importantly, this review also gives a wider perspective of the consequences of lipid imbalances in the cell, potentially leading to cancer, metabolic disease and neurological disease. In the case of the latter, there is growing evidence not only that the local lipid environment of organelles such as the endoplasmic reticulum (ER) and mitochondria is an essential feature of normal neuronal cell function, but also that the specific shape, distribution and way in which organelles make contact with one another is contributory to diseases such as hereditary spastic paraplegia [10]. Understanding the mechanisms of how specific lipids localise to subdomains of organelles is an essential aspect of cell biology [11], and it is exciting to see new methodologies emerging, for example Raman microscopy [12], which have the potential to provide high-resolution lipid profiling in live cells. 

Another article that highlights the link between organelle morphology, specifically, the nuclear envelope, and disease, is provided by Alvarado-Kristensson and Rossello [4]. The authors describe not only the physical shape of the nuclear envelope, the underlying nuclear lamina and the dramatic rearrangements that they undergo during cell division, but also roles in cell signalling. They go on to discuss how this organelle links to cell migration and metastasis in cancer, which, again, is a theme receiving increased attention [13,14]. Four of the articles in this special issue address aspects of the morphology and function of the ER, a compartment that is contiguous with the nuclear envelope. The ER is an extremely complex organelle, involved in a wide range of biochemical activities, most notably protein glycosylation and folding, detoxification and calcium storage. The ER faces a number of challenges, not least the variety of cargo that it needs to assemble and ensure that is correctly folded prior to export. This aspect is dealt with in a detailed review from the group of Manuel Muniz, who describe the biogenesis of a particular family of cargo molecules, i.e., glycosylphosphatidylinositol (GPI)-anchored proteins (GPI-APs), which ultimately function at the plasma membrane [3]. However, the issue facing many highly active secretory cells is how to deal with accumulations of misfolded proteins in the ER. The mechanism typically invoked is the so-called ‘unfolded protein response’ (UPR), which allows cells to activate additional mechanisms such as the upregulation of expression of molecular chaperones to boost the folding capacity of the organelle [15]. The activation of the UPR in a variety of immune cells has been reported [16], and in this special issue, details are given with respect to the UPR activation pathways in T-cells and the consequences for the immune system [9]. 

The theme of ER morphology in disease and infection is returned to in a review by Arakawa and Morita [5]. This organelle occupies a significant volume in many cell types, and, as such, is a natural target for exploitation by viruses that need a source of membranes for their assembly in infected cells. Indeed, recent articles have highlighted that not only is it the ER membrane that is used by viruses, but that, in fact, certain viruses such as hepatitis B [17] and rotavirus [18] specifically subvert the ER export machinery, as discussed in [3], to their ends. Arakawa and Morita provide a detailed review of how the ER is used as a replication organelle for the flaviviruses, a group that includes the dengue, Zika and tick-borne encephalitis viruses, among others [5]. What is striking is that these viruses target specific phospholipid subdomains of organelles, as well as having the ability to utilise endogenous ER-shaping proteins, such as the reticulons, to remodel the ER into structures that suit their needs.

The Golgi complex is another organelle that features prominently in the theme of modulation of endomembrane architecture. The Acyl-CoA-Binding Domain-Containing 3 (ACBD3) scaffolding proteins operate to form functional membrane microdomains at the Golgi complex, binding to palmitoyl-CoA as well as the Golgi matrix proteins giantin, golgin-160 and golgin-45. Yue and colleagues provide an overview of ACBD3 function in normal cells, and highlight how this protein family can be exploited by viral and bacterial proteins in infected cells [7]. The Golgi complex, as a central organelle in the cell, is also considered within the context of autophagy, and specifically, through the activity of the small GTPase Rab33b. In a review by Morgan and colleagues [2], consideration is given not only to Rab33b function in membrane trafficking through the Golgi complex, but also to the growing evidence [19,20] that this Golgi resident plays additional roles in autophagy.

In summary, as we build our knowledge of how the endomembrane system is regulated and modulated at the molecular level, it is becoming increasingly clear that not only is this information important for our continued dissection of cell function, but that it forms a vital component in furthering our understanding of disease. I am grateful to all the authors who have contributed to this special edition, which I hope showcases the variety of cellular events where membrane architecture and remodelling are important phenomena, and which, in turn, contributes to the physiology of cell behaviour. 

## Figures and Tables

**Table 1:** Contributions to the Special Issue “Molecular Regulation of the Endomembrane System”.

Authors	Title	Membranes of Focus	Type
Kusanaga et al. [1]	Zinc Attenuates the Cytotoxicity of Some Stimuli by Reducing Endoplasmic Reticulum Stress in Hepatocytes	ER, lysosomes, autophagosomes	Original research
Morgan et al. [2]	Multitasking Rab Proteins in Autophagy and Membrane Trafficking: A Focus on Rab33b	Golgi, autophagosomes	Review
Lopez et al. [3]	Endoplasmic Reticulum Export of GPI-Anchored Proteins	ER	Review
Alvarado-Kristensson and Rossello [4]	The Biology of the Nuclear Envelope and Its Implications in Cancer Biology	nucleus, nuclear envelope	Review
Arakawa and Morita [5]	Flavivirus Replication Organelle Biogenesis in the Endoplasmic Reticulum: Comparison with Other Single-Stranded Positive-Sense RNA Viruses	ER	Review
Casares et al. [6]	Membrane Lipid Composition: Effect on Membrane and Organelle Structure, Function and Compartmentalization and Therapeutic Avenues	Multiple	Review
Yue et al. [7]	Acyl-CoA-Binding Domain-Containing 3 (ACBD3; PAP7; GCP60): A Multi-Functional Membrane Domain Organizer	Golgi, mitochondria	Review
Michie et al. [8]	Two Sides of the Coin: Ezrin/Radixin/Moesin and Merlin Control Membrane Structure and Contact Inhibition	plasma membrane	Review
Kemp and Poe [9]	Stressed: The Unfolded Protein Response in T Cell Development, Activation, and Function	ER	Review
